# GANscan: continuous scanning microscopy using deep learning deblurring

**DOI:** 10.1038/s41377-022-00952-z

**Published:** 2022-09-07

**Authors:** Michael John Fanous, Gabriel Popescu

**Affiliations:** 1 Quantitative Light Imaging Laboratory, Beckman Institute for Advanced Science and Technology, University of Illinois at Urbana-Champaign, Urbana, IL 61801 USA; 2 Department of Bioengineering, University of Illinois at Urbana-Champaign, 306 N. Wright Street, Urbana, IL 61801 USA; 3 Department of Electrical and Computer Engineering, University of Illinois at Urbana-Champaign, 306 N. Wright Street, Urbana, IL 61801 USA

**Keywords:** Optical sensors, Phase-contrast microscopy

## Abstract

Most whole slide imaging (WSI) systems today rely on the “stop-and-stare” approach, where, at each field of view, the scanning stage is brought to a complete stop before the camera snaps a picture. This procedure ensures that each image is free of motion blur, which comes at the expense of long acquisition times. In order to speed up the acquisition process, especially for large scanning areas, such as pathology slides, we developed an acquisition method in which the data is acquired continuously while the stage is moving at high speeds. Using generative adversarial networks (GANs), we demonstrate this ultra-fast imaging approach, referred to as GANscan, which restores sharp images from motion blurred videos. GANscan allows us to complete image acquisitions at 30x the throughput of stop-and-stare systems. This method is implemented on a Zeiss Axio Observer Z1 microscope, requires no specialized hardware, and accomplishes successful reconstructions at stage speeds of up to 5000 μm/s. We validate the proposed method by imaging H&E stained tissue sections. Our method not only retrieves crisp images from fast, continuous scans, but also adjusts for defocusing that occurs during scanning within +/− 5 μm. Using a consumer GPU, the inference runs at <20 ms/ image.

## Abstract (web summary)

In order to speed up the microscopy acquisition process, we developed a method, termed GANscan, in which videos are recorded as the stage is moving at high speeds. Using generative adversarial networks (GANs), we achieve 30x the throughput of stop-and-stare systems.

## Graphical abstract

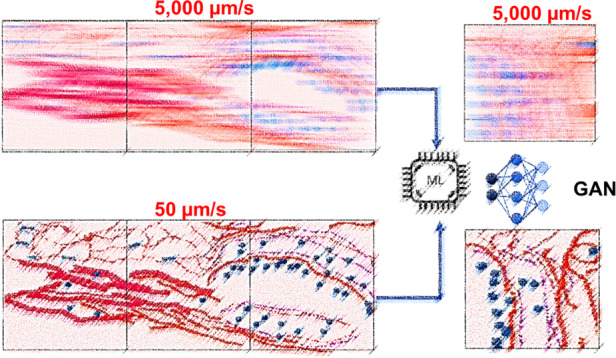

## Introduction

Numerous microscopy applications require large fields of view (FOV), including digital pathology^1^, micro-mirror and biosensor assembly^2^, and in vivo imaging^3^. Acquisition time demands are a major bottleneck to fixing modest or partially filled FOVs in standard microscopy techniques. Improvements in both hardware and computation are thus actively sought to push the efficiency of optical measurements beyond traditional boundaries. Accelerating either image acquisition or analysis can have drastic benefits in diagnostic assessments and has been shown to provide critical advantages in cell detection^4^, disease screening^5^, clinical studies^6^ and histopathology^7,8^.

In standard microscope systems, the objective lens dictates the resolution and field-of-view (FOV), forcing a trade-off between the two parameters. In commercial whole slide scanners, the FOV is extended through lateral scanning and image mosaicking. Most forms of microscopy require serial scanning of the sample region, which slows down measurement acquisitions and diminishes the temporal resolution.

There are three classes of strategies used in traditional microscopy for slide-scanning. The first technique uses the so called “stop-and-stare” style, which entails sequentially moving the sample across a scanning grid, pausing the stage, and exposing the camera for discrete recordings. This tactic generates high-quality images as a result of long measurement durations, but is not especially time-efficient^9^. A second technique involves illuminating a moving sample with bursts of light that help circumvent the motion blur, which would otherwise compromise the image resolution. As a result of the short exposure times with this method, the resulting images have a relatively poor signal-to-noise ratio (SNR)^9^. Thus, there is a cost to optimizing image clarity or acquisition speed in these approaches. Third, there are line scanning^10^ and time-delay integration (TDI)^11^ methods, which use 1D sensors, where a camera vertically handles continuous signals line by line to reduce read-out time and increase SNR. However, even the latest versions of these instruments require specialized imaging equipment and readout methods^12,13^.

Different imaging methods have been proposed to improve the throughput of scanning-based microscopy techniques, such as multifocal imaging^14^ and coded illumination^9^. Computational methods of microscopy imaging^15–19^, such as ptychography, which scans and fuses portions of spatial frequencies, can produce large FOVs with resolutions that surpass the objective’s diffraction limit. However, these solutions end up either complicating the microscopy system configuration, deteriorating the image quality, or extending the post-processing period. Additionally, iterative algorithms that are used in Fourier ptychography to reconstruct an image from a sequence of diffraction patterns often suffer from convergence issues^20^.

The mechanical specifications of the scanning stage, rather than the optical parameters of the microscope, generally hinder the throughput performance of WSI systems^21^. The space-bandwidth product (SBP), which is the dimensionless product of the spatial coverage (FOV) and the Fourier coverage (resolution) of a system, can quantify the information across an imaging system^22^. Enhancements to the SBP have been the objective of various innovations in imaging techniques^23–28^, but typically require either specialized hardware or time-consuming post-processing.

The advent of accessible deep learning tools in recent years has led to a new host of strategies to address lingering microscopy challenges^27^, including super-resolution imaging^29^, digital labeling of specimens^30–37^, Fourier ptychography microscopy^26^, and single-shot autofocusing^38^, among others^39^. These methods, which take advantage of recent breakthroughs in deep learning, need no modification to the underlying microscopic gear and produce faster and more comprehensive imaging results than traditional image reconstruction and post-processing algorithms. Generative adversarial networks (GANs), which comprise two opposing networks competing in a zero-sum dynamic, have been especially prominent in image-to-image translation tasks, due in large part to their outstanding execution of pixel-to-pixel conversions^31,40^.

In this work, we propose a computational imaging technique, termed GANscan, which employs a GAN model to restore the spatial resolution of blurred videos acquired via continuous stage scanning at high speeds using a conventional microscopy system. Our method involves continuously moving the sample at a stage speed of 5000 μm/s and an acquisition rate of 30 frames per second (fps). This acquisition speed is on par with the state-of-the-art TDI technology of 1.7–1.9 gigapixels in 100 s^11,13^. However, unlike TDI, our approach is using standard optical instrumentation, which lowers the threshold for broad adoption in the field.

In contrast to other high-throughput imaging endeavors, GANscan adds no complexity to the hardware, with single frame restorations that can be computed in a matter of milliseconds. The results of this novel technique demonstrate that basic modifications in measurements, coupled with artificial intelligence (AI), can provide the framework for any rapid, high-throughput scanning operation.

This paper is structured as follows: first, we present the workflow for continuous imaging microscopy in both slow and fast acquisitions. Second, we describe the theory behind blur motion artifacts and why deconvolutions are limited in restoring the spatial bandwidth of control images. Third, we discuss the imaging procedures and registration of slow-moving samples with the motion-smeared ones. Fourth, the parameters of the GANscan network are explained, as well as the data processing techniques prior to model training. Lastly, reconstruction performances are evaluated using an unseen test set, including a test set from different patients, which is also compared against stop-and-stare controls and deconvolutions using standard image metrics.

## Results

### Workflow

**Fig. 1:**
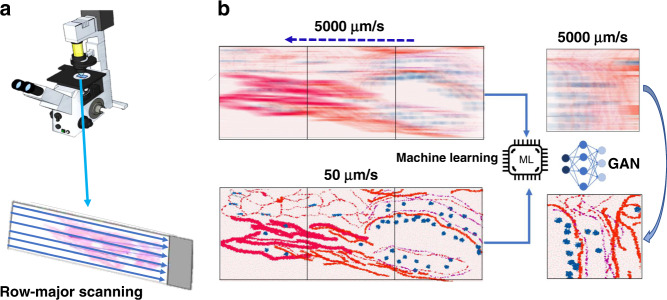
GANscan: setup and concept. **a** Scanning stage of the AXIO observer Zeiss microscope with an example slide showing the row-major continuous scanning direction. **b** Motion blurred reconstruction scheme using a slow-moving stage as the control for GAN training

Figure 1 depicts the workflow of our approach. To demonstrate the benefits of this technique, we imaged a large sample of a pathological slide of a ductal carcinoma in situ (DCIS) biopsy, covering roughly half a standard microscopy slide area (~30 mm × 15 mm), as well as an unstained blood smear. All slides studied in this work were divorced from patient statistics, with consent from Carle and Christie Clinic in Urbana, Il, and their use was approved by the institute review board at the University of Illinois at Urbana–Champaign (IRB Protocol Number 13900). Both slides were scanned in a row-major configuration, capturing movies across the slide horizontally (Fig. 1a). There were no modifications to a standard commercial microscope (Axio Observer Z1, Zeiss), and the only adjustments in the measurement were the speed of the stage and the continuous recording of the camera. In order to obtain ground truth images for training, the same rows were captured at a slow (50 μm/s) stage speed and at the same exposure time of 2 ms. Once pairs of sharp and defocused images were assembled through Pearson correlations, a GAN network was trained to enable restoring unseen motion blurred micrographs (Fig. 1b).

### Theory

**Fig. 2:**
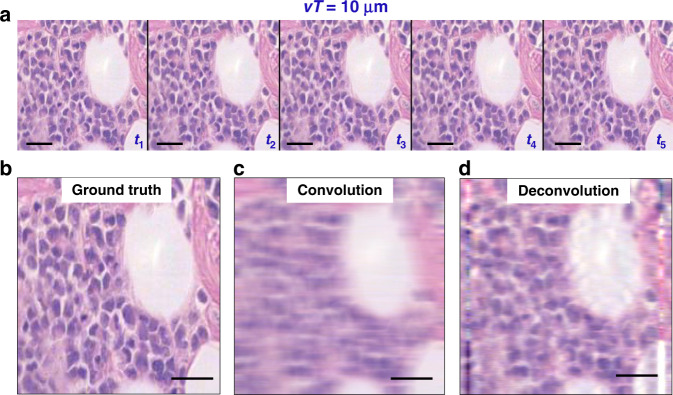
Comparison of ground truth with convolved and deconvolved images. **a** A few frames from a video taken with a stage speed of 50 μm/s, with time labels indicating a slow forward movement. **b** Middle image of the sharp sequence **a** used as ground truth. **c** The convolution of **b** with the blur function. **d** The deconvolution of **c** with the blur function. Scale bar 25 µm

At rest, let the image be $I(x,y)$ During the sample translation, the translated image, $\underline I$, has the following time dependence:

1 $$\underline I (x,y;t) = I(x + vt,y)$$

where *v* is the stage speed. Considering the camera integration time T, the “blurred” detected frame is then:

2 $$\begin{array}{*{20}{c}}{\underline I (x,y) = \mathop {\int}\nolimits_{ - T/2}^{T/2} {I(x + vt,y)dt} }\\\qquad\qquad\qquad { = \mathop {\int}\nolimits_{ - \infty }^\infty {I(x + vt,y){{{\mathrm{{\Pi}}}}}\left( {\frac{t}{T}} \right)dt} }\end{array}$$

where ${{{\mathrm{{\Pi}}}}}\left( {{\textstyle{t \over T}}} \right)$ is the 1D rectangular function of width *T*. The integration is the sum of the frames accumulated during the acquisition time *T* (Fig. 2a).

Using the central-ordinate theorem:^41^

3 $$\underline I (x,y) = \left. {\Im _t\left\{ {I(x + vt,y)\prod \left(\frac{t}{T}\right)} \right\}} \right|_{\omega = 0}$$

where ω is the angular frequency. Since $I(x + vt,y) = I[v(t + \frac{x}{v}),y],$ the temporal Fourier transform reads

4 $$\begin{array}{l}I(x + vt,y) = I\left[ {v\left( {t + \frac{x}{v}} \right)} \right]\\ \quad \quad \quad \quad \leftrightarrow I\left[ {\frac{\omega }{v},y} \right]e^{ - i\frac{{\omega x}}{v}}\end{array}$$

where ↔ indicates the Fourier transformation.

Using the convolution theorem^42^, Eq. 3 can be rewritten as:5

In Eq. 5, we recognize a Fourier transform of a product, which yields the following convolution operation,6where  indicates the convolution operator over the variable *x/v*, which has dimensions of time. This result captures the physical description of the image *spatial* blurring as the result of a *temporal* convolution operation. Thus, the smeared image is the sharp image convolved along the direction of the scan by a rectangular function, which has a width proportional to the acquisition time. For a scanning speed of *v* = 5000 *μm*/*s* and *T* = 2 *ms*, *vT* = 10 *μm*. This corresponds to a length roughly twenty times the diffraction resolution of our imaging system.

### Deconvolution

We performed the 1D *deconvolution* on our acquired images, thus, inverting the effect of Eq. 5, and used the results as the standard of comparison for the deep learning results. These deconvolutions were evaluated by first establishing the best match through the ‘convolve’ filter in ImageJ, and then using the same line dimension in MATLAB with the ‘deconvblind’ function. This tool deconvolves an image via the maximum likelihood algorithm and a starting estimate of the point-spread function (PSF), which in our case is a single row of 47 pixels of value 1.

A sample frame of the biopsy and its convolution with the line of the blur width are shown in Fig. 2b, c, and deconvolving again produces the original frame but with compromised high spatial frequencies (Fig. 2d). The artifacts of lines along both edges of the image are a result of the filter brushing against the boundaries of the image. The deconvolution operation succeeds at shrinking features horizontally to restore their true width. However, the image still suffers from poor overall resolution, due in part to the higher spatial frequencies being permanently lost through the convolving effect of imaging a rapidly moving sample. This shortcoming is our principal motivation of employing deep learning techniques to predict the standard spatial bandwidth.

### Image pair registration

**Fig. 3:**
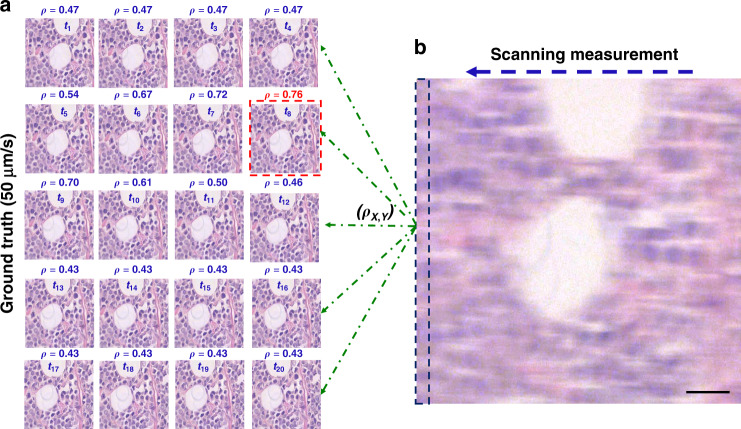
Sharp and blurry image pair registration scheme. **a** Registration of images through maximum Pearson coefficient between sharp frames at 50 μm/s and **b** the blurry one at 10,000 μm/s. The scanning measurement is compared with each sharp contender (green dashed lines) and the resulting coefficient score is listed above each image. The best match is delineated in red, indicating the sharp image bearing the overall highest resemblance to the streaked measurement. The dashed rectangle at left edge of **b** outlines the extra margin of features contained in a blurry image (10 μm)

In order to prepare pairs of blurred and sharp images for training, consecutive sharp images in the fast videos were matched to their motion-smeared counterparts by evaluating the maximum Pearson correlations in a set of slightly shifted clear images (Fig. 3, S1). The “ground truth” images were captured at a stage speed of 50 μm/s, which, at the acquisition time of 2 ms results in a blur size of 0.1 *μ*m, *i.e*., below the diffraction limit of our system. As a result, there are approximately 100 frames in the sharp videos for each image in the 5,000 μm/s, motioned blurred videos, as shown in Fig. 3a.

Evaluating the Pearson correlation between the input (smudged) frame and a series of potential ground truth frames produces values ranging from 0.4 to 0.76. The frame associated with the highest Pearson correlation value was selected as the ground truth. It should be noted that the rapidly captured images expose the camera to a larger field of view than the slowly-captured ones by the length of the blur, which is 10 *μ*m for a scanning speed of 5000 μm/s. This difference is delineated in Fig. 3b.

In order to confirm the reliability of the ground truth images obtained at 50 μm/s, standard stop-and-stare images were also acquired for comparison as part of a test set. This was achieved by serially scanning images with a lateral shift of 1 μm, mimicking the distribution of slowly moving images but fully halted. It was necessary to capture sufficient images in order not only to perfectly register the stop-and-stare images with the 50 μm/s images, again using a Pearson correlation computation, but also with the blurry images. As shown in Fig. S2, the stop-and-stare images look identical to the 50 μm/s images, with SSIM values upwards of 0.9. Variability in values is possibility indicative of noise inherent in the images. These are further compared with the moving ground truths against the reconstructed images, discussed below.

### Generative adversarial network (GAN)

**Fig. 4:**
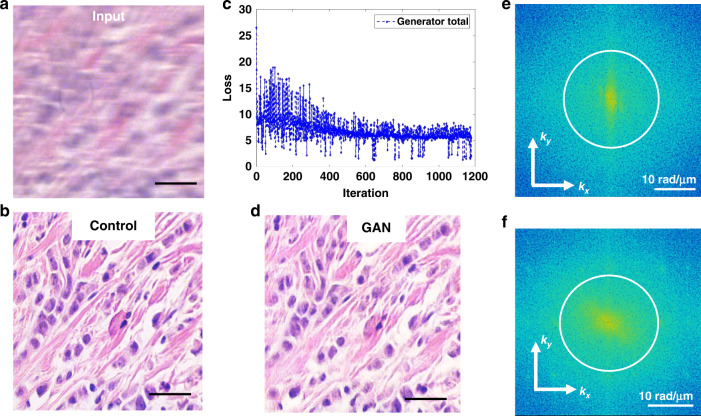
Network performance with frequency analysis. **a** Training example of the blurry input, **b** and control, **c** with the loss plot. **d** The generated result for a training instance. **e** The power spectra of the input and **f** of the result with a circle outlining the diffraction spot. Scale bar in **a**, **b**, and **d** 25 µm

Once the registered pairs were assembled, they were cropped and resized to dimensions of 256 × 256 × 1-3 (3 RGB color channels for brightfield and 1 channel for phase contrast images) for faster computation, with 1050 images earmarked for training and 50 reserved for testing for both sample types. The architecture of the model consists of a generator U-net with eight encoding and decoding layers, and a four downchannel discriminator, all displayed in Fig. S3. As shown in Fig. 4, the network input is the motion-blurred image and the control is the slowly scanned, sharp image. Since the slide was scanned in a row-major style, the margin of additional field of view is always on the same side, which is likely to help the network to undo the motion distortion. The GAN model was trained for over 200 epochs (Fig. 4c) until the loss function plateaued. Our results indicate that running the model on the training set produces nearly perfect restorations (Fig. 4d). The spatial power spectrum of the input image (Fig. 4e) clearly shows a smaller range of higher spatial frequencies than that of the restored image (Fig. 4f). Interestingly, the power spectrum of the input image has higher spatial frequencies along the vertical axis as a result of the smearing produced along the x-axis, whereas the power spectrum of the restored image is broader and more isotropic.

A separate model for repairing out-of-focus images was computed using 2420 training image pairs (taken from two different slides), with nearly half of the blurry set captured at four different levels of focus: −10 μm, −5 μm, +5 μm, and +10 μm. The rest of the parameters were the same as the models described above, with the ground truth images all being in perfect focus.

### Performance testing

**Fig. 5:**
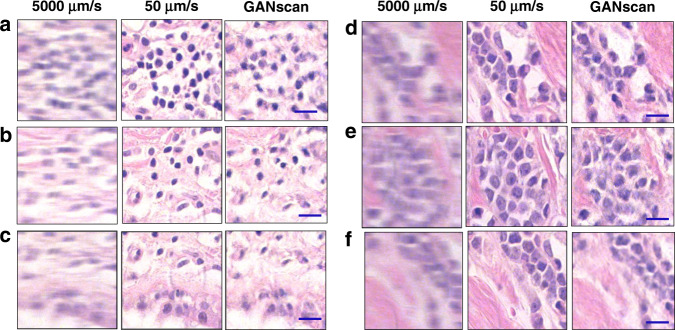
Examples of test results. **a**–**f** Brightfield test set conversion of 5000 µm/s with control of 50 µm/s. Scale bar 5 µm

**Fig. 6:**
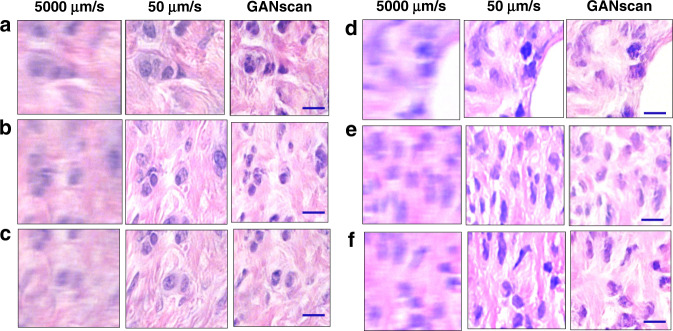
Examples of test results from unseen pathology slides. **a-c**, **d**–**f** Test set conversion of 5000 µm/s with control of 50 µm/s from two patients not included in the training dataset. Scale bar 5 µm

Once the training was complete, the model was tested on 50 *unseen* images of the same slide of the dataset, and 160 images from slide corresponding to a different patient, as shown in Figs. 5 and 6, respectively. 50 unseen blood smear images were also reconstructed, a sample of which is shown in Fig. S4. The network does an effective job at restoring the high spatial frequencies of epithelial and stromal (fibrous) areas in biopsy samples, as compared to the line deconvolutions (Fig. S5). Since the cellular and fibrous areas are recovered with such high fidelity, the diagnostic information in the tissue images is maintained in full. In terms of numerical assessments, the first biopsy test sets achieved an average structural similarity index measure (SSIM) of 0.82 and a mean peak signal-to-noise-ratio (PSNR) of 27 when calculated against their controls. For the same dataset, the deconvolution results gave inferior results of SSIM and PSRN of 0.71 and 26, respectively. The biopsy test set corresponding to a different patient achieved a similar average structural similarity index measure (SSIM) of 0.83 and a mean peak signal-to-noise-ratio (PSNR) of 26 when calculated against their controls, proving that the technique is applicable to samples entirely separate from of the training data (Fig S6). For the same dataset, the deconvolution results again gave inferior results of SSIM and PSRN of 0.77 and 25, respectively.

The same metrics were also calculated against stop-and-stare ground truths set. Fig. S7 shows that there is no statistically significant difference between the values using the stop-and-stare and the 50 μm/s controls, indicating that the pairing strategy is valid.

Another way the results were evaluated was using line sections and plot profiles. Fig. S8 shows a sample biopsy image in all three modes with their power spectra and line sections. The brightfield plot profiles show a strong overlap between the slow and reconstructed images, whereas the blurry image has a line profile that is smeared and diminished in intensity. In the frequency domain, the slow and reconstructed images show broader and higher frequencies as compared to the power spectrum of the blurry image, as expected.

The blood smear phase contrast images were reconstructed with similar success (Figs. S8 and S9). GANscan does an effective job at replicating a standard phase contrast image from a highly blurred input. Although some of the cell edges are not as smooth and round as in the control data, there is rarely any hallucination of new cell boundaries. In this case, the test sets achieved a slightly lower average structural similarity index measure (SSIM) of 0.73 and a mean peak signal-to-noise-ratio (PSNR) of 27 when calculated against their controls. For the same dataset, the deconvolution results gave inferior results of SSIM and PSRN of 0.66 and 26, respectively. A possible reason for the lower GANscan values with phase contrast microscopy may be that only the edges and halo of the blood cells contain any signal, causing a more severe blur in these images. As well, a single grayscale channel provides less information and context for the network to deal with.

**Fig. 7:**
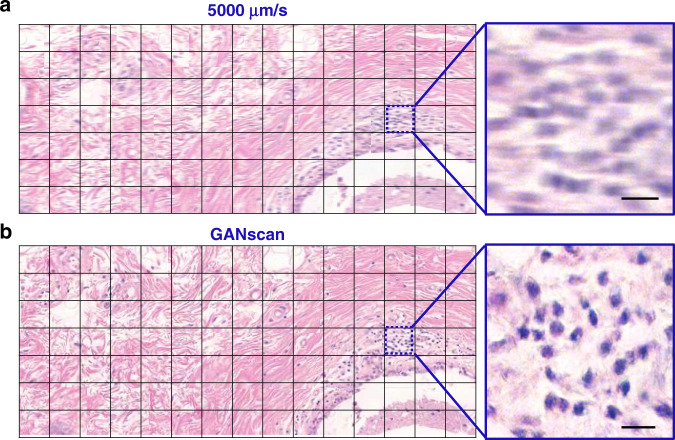
Comparison of motion-blurred and reconstructed tissue mosaics. **a** Stitch of the motion blurred images, **b** as well as the reconstructed GANscan, showing a large area of a breast biopsy, with respective zoom-ins. Scale bar 5 µm

Large mosaics of the motioned blurred biopsy images were also reconstructed (Fig. 7) by concatenating the images horizontally and vertically in their respective scanning order, producing a 7 × 15 stitch of roughly 3 mm × 1.5 mm in size. The difference in clarity is much less apparent with such a large FOV, but at a closer look it is evident there is significant improvement in the overall distinction of features. Stitches for 4,000 μm/s were also made for comparison (Fig. S10).

### Adjusting out-of-focus images

**Fig. 8:**
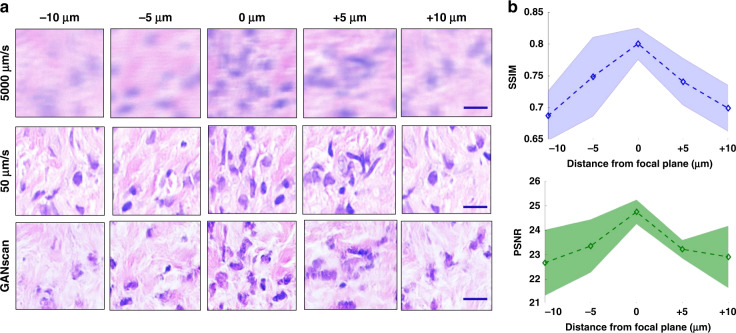
Relationship between focus offsets and motion-blur restorations. **a** Example images from 5 different points of focus in fast, slow and reconstructed modes. **b** SSIM and PSRN values at the different points in the focal plane with shading representing the standard deviation across the 150-image (30 per plane) test set. Scale bar 5 µm

In order to assess the ability of GANscan to repair defocused images, the test set from a different patient was captured at 5000 μm/s at the plane of focus, but also at −10 μm, −5 μm, +5 μm, and +10 μm. This idea was inspired by previous work addressing autofocusing methods using GAN models^43^. Figure 8a shows a sample of images in all three modes at various levels of focus. They are not corresponding FOVs, as it is not possible to perfectly match different focal scans of blurry images. As it can be seen, the reconstructed images become progressively worse with increasing distance from the focal plane. Figure 8b shows the SSIM and PSNR curves for the whole test set with standard deviation margins from 30 instances (images) per level of focus. It is clear that the SSIM and PSNR start dipping as the image loses focus, from over 0.82 and 25, respectively, dropping to below 0.65 and 22 at the +/−10 μm mark.

## Discussion

We presented a high-throughput imaging approach, GANscan, which employs continuous motion deblurring using labelled GAN reconstructions. Through both theoretical and experimental analysis, we have demonstrated the applicability of our method to brightfield and phase contrast microscopy on tissue slides. Our results indicate that GAN models provide, in combination with greater stage speeds, up to 30x faster acquisition rates than in conventional microscopy. This throughput is superior or on par with the state-of-the-art rapid scanning techniques, which in turn use nonstandard hardware. GANscan requires no specialized equipment and generates restored images with successfully removed motion blur. Of course, should a camera with a higher frame rate be used, the stage speed can be scaled up proportionally. Further, our proposed deep learning deblurring method produces high-quality reconstructions which restores the high frequency portions of the tissue and cells, as opposed to deconvolution operations.

Such a methodology will not only provide a drastic benefit in the clinical setting to pathologists for diagnosis of cancer in biopsies and cell abnormalities in blood smears, but at the research level as well, including cell cultures of large dimensions. Future work should address achieving similar results with different microscope modalities, such as fluorescence and quantitative phase imaging.

## Material and methods

### Image acquisition

Images were acquired with a commercial microscope (Axio Observer Z1, Zeiss) in brightfield and phase contrast settings and a Point grey color camera, using a Zeiss EC Plan-Neofluar 40x/0.45 NA objective. The samples were a ductal carcinoma in situ (DCIS) breast tissue biopsy and an unstained blood smear of a healthy patient. The stage speed and coordinates were precisely manipulated using the Zeiss MTB (MicroToolBox) software, and the camera settings, such as shutter time (2 ms), frame rate (30 pfs), and gain (8 dB), were selected using the Grasshopper GRAS- 2054 C software. For stitching images, a vertical step size of 200 µm was used, and horizontal videos were acquired for 1 minute at the slow speed of 50 µm/s to ensure the correspondence of 15 horizontally adjacent frames in the video captured at 5000 µm/s. The videos of each row at the accelerated stage speed was 0.6 seconds. After the image acquisition, off-line processing involved image registration of blurry and sharp images through MATLAB with Pearson correlation estimates. For the 5000 µm/s datasets, we extracted 256 × 256 crops from paired images to create a training volume of 1050 image pairs.

We performed deconvolutions on each input test image and compared them with GANscan results, as shown in Fig. S2. The mean SSIM of the GANscan biopsy images is 0.82, while the deconvolved images had an SSIM of 0.73, when compared to the same control images. The mean SSIM of the GANscan phase contrast images is 0.73, while the deconvolved images had an SSIM of 0.66, when compared to the same control images. PSNR values were also calculated with GANscan outperforming deconvolutions 27 to 26 for both image types. All analysis was performed in MATLAB.

### Machine learning

The conversion of motion blurred micrographs to sharp images was accomplished using the conditional generative adversarial network (GAN) pix2pix (Fig. S4)^44^. The same parameters and steps were applied for training both the brightfield and phase contrast images. The only difference was the number of channels of the images, with three for the RGB colored images and one for the grayscale phase contrast blood smear images.

1050 blurry and sharp brightfield image pairs were passed through the network for the first model, 2420 for the second with different focus levels. Original dimensions of the micrographs were 600 × 800 pixels. These were cropped and resized to 256 × 256 pixels before being trained on. The learning rate of the generator’s optimizer was 0.0002 and the minibatch size was set to 1. In this network, a generator (G*)* is trained to produce outputs that cannot be distinguished from ground truth images by a trained adversarial discriminator, *D*, which is designed to perform as well as possible at detecting the generator’s incorrect data^44^. The GAN loss is one where *G* works to minimize the value while an adversarial *D* attempts to maximize it:

7 $$L_{cGAN}\left( {G,D} \right) = E_{x,z}\left[ {\log D\left( {x,y} \right)} \right] + E_{x,z}\left[ {\log \left( {1 - D\left( {x,G\left( {x,z} \right)} \right)} \right.} \right]$$

Where *E*_*x*, *z*_ is the anticipated value of all real and fake instances, x is the image, and z is the generated random noise. An L1 loss is then combined with this to generate the discriminator’s total loss function.

In order to confirm the accuracy of the translated images, we tested the model on 50 unseen images and 160 different patient images. Training was performed over 200 epochs, with datasets that were augmented beforehand through rotations and mirroring. Overall, the training took 7 h for each model, and the inference required less than 20 ms per image (256 × 256 pixels).

## Supplementary information

Supplementary Information for GANscan: continuous scanning microscopy using deep learning deblurring

## Data Availability

All data required to reproduce the results can be obtained from the corresponding author upon a reasonable request.

## Code availability

All the code required to reproduce the results can be obtained from the corresponding author upon a reasonable request.
