# Supplementary material for: GANscan: continuous scanning microscopy using deep learning deblurring
Source: Light Sci Appl. 2022 Sep 7;11:265. doi: 10.1038/s41377-022-00952-z (PMC9452654; doi:10.1038/s41377-022-00952-z)
Supplement: Supplementary file 1 — Supplementary Information for GANscan: continuous scanning microscopy using deep learning deblurring [file 41377_2022_952_MOESM1_ESM.docx]

**Supplementary Information for**

**GANscan: continuous scanning microscopy using deep learning deblurring**

*Michael Fanous^1,2^ and Gabriel Popescu^1,2,3^*

1. Quantitative Light Imaging Laboratory, Beckman Institute for Advanced Science and Technology, University of Illinois at Urbana-Champaign, Urbana, Illinois 61801, USA
2. Department of Bioengineering Department of Bioengineering, University of Illinois at Urbana-Champaign, 306 N. Wright Street, Urbana, IL 61801
3. Department of Electrical and Computer Engineering, University of Illinois at Urbana-Champaign, 306 N. Wright Street, Urbana, IL 61801, USA

**S1. Image registration:** Consecutive sharp images in rapid movies were matched to their motioned deformed counterparts by calculating the maximum Pearson correlations in a collection of slightly shifted sharp photos. The blurred and sharp images were thus paired in a sequence of comparisons (Figs. 3, S1). The "ground truth" photos were obtained at a stage speed of 50 µm s^-1^, resulting in a blur size of 0.1 m at a 2 ms acquisition time, which is below our system's diffraction limit. As a result, each image in the motioned blurred videos has around 100 frames in the sharp videos, as seen in Figure 3.


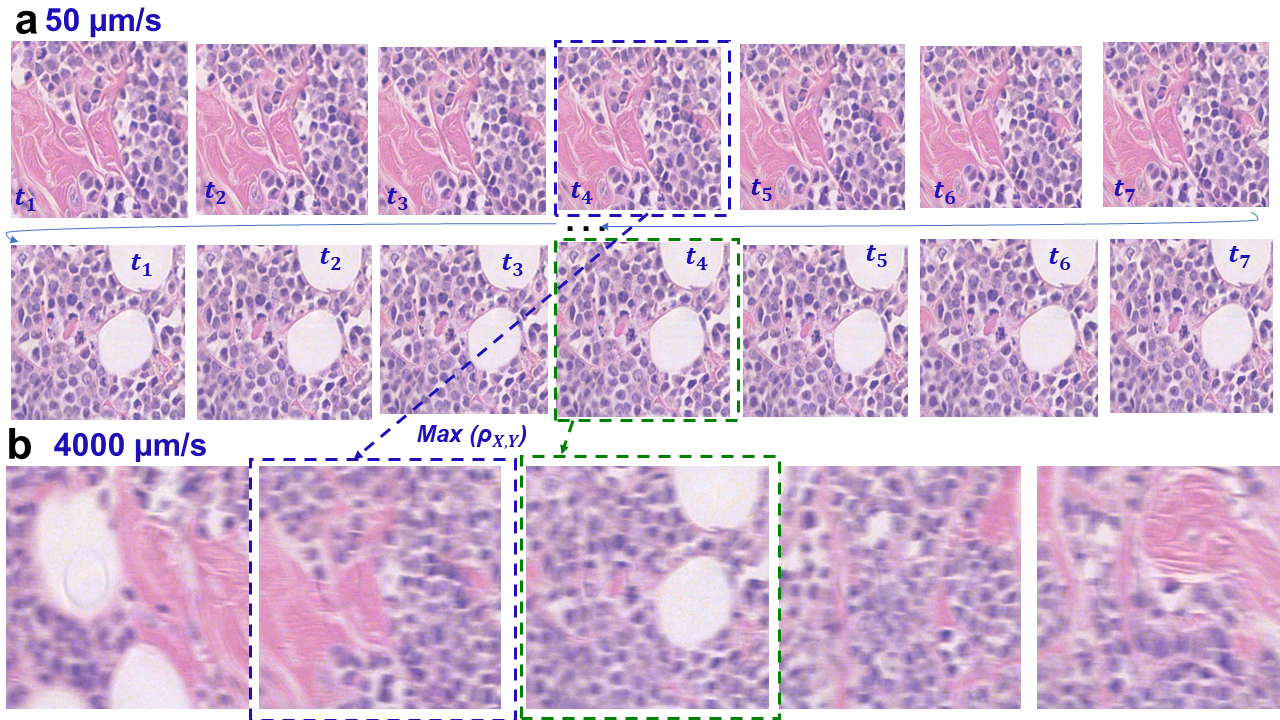


Figure S1. Registration of images through maximum Pearson coefficient between **a** sharp frames at 50 μm/s and **b** blurry ones at 4000 μm/s**.** The images are labelled with time stamps indicating a forward moving slow stage and best matches indicated with dashed lines.

**S2.** **Stop-and-stare comparisons:** in order to assess the reliability of the ground truth images captured at 50 µm s^-1^, standard stop-and-stare images were also acquired for comparison. This was done by scanning images with a lateral increment of 1 mm, duplicating the series of slowly moving images but completely halted. It was essential to capture enough images in order not only to perfectly match the stop-and-stare images with the 50 µm s^-1^ images, again using a Pearson correlation calculation, but also with the blurry images. As shown in Figure S2, the stop-and-stare images appear identical to the 50 µm s^-1^ images, with SSIM values upwards of 0.9. Variability in values is possibility indicative of noise inherent in the images.


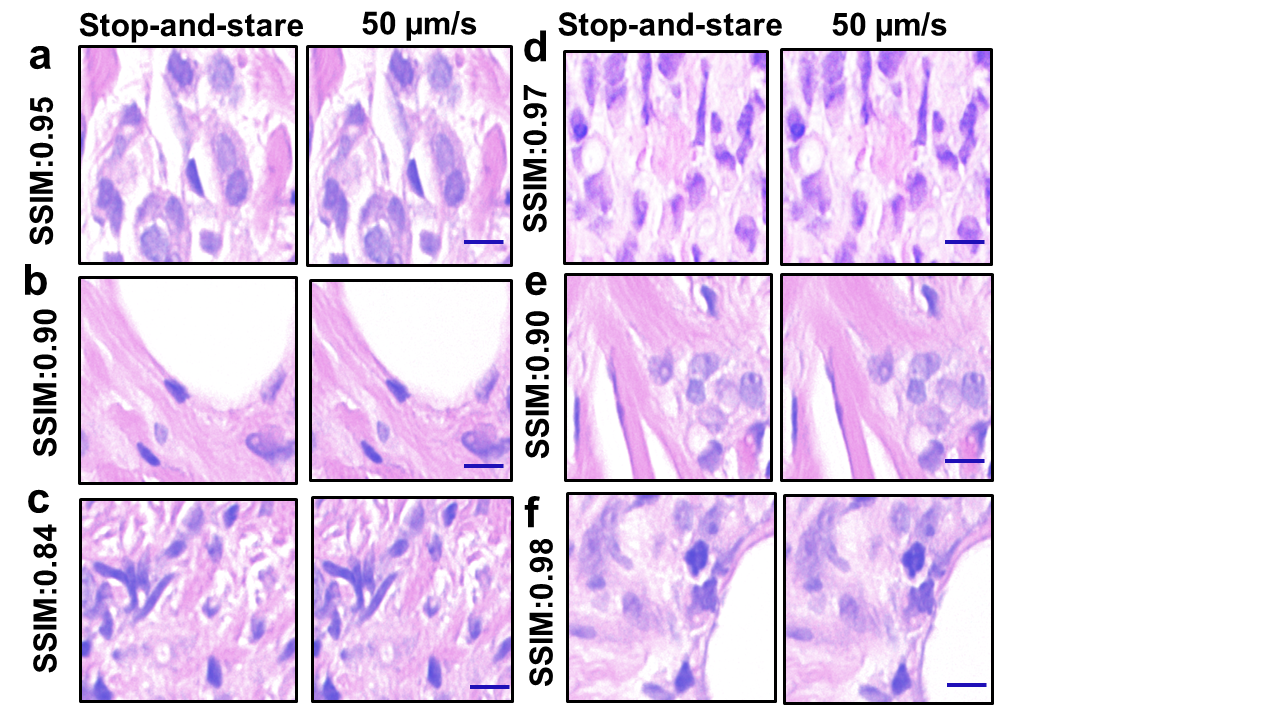


Figure S2. **a-f** Example comparisons of stop-and-stare images with images recorded at 50µm/s with SSIM values. Scale bar **5 µm**.

**S3. Model architecture and training strategies:** For the deep convolutional neural network, we picked a variant of a generative adversarial network (GAN) that has shown great performance on image translation tasks previous works[1-5]. The network contains two competing models, a generator, and a discriminator (Figure S1). The generator is a U-Net, which has eight layers of encoding and decoding. The discriminator has 4 stages of encoding before making a decision on the authenticity of the data instance [6].

The model was trained with batches of images of size 256 × 256 x 1-3 pixels, cropped from 600x800x1-3 images from our training set. The batch size was set to 2. The learning rate was set to 0.0002 and the maximum number of epochs was set to 200.

The model was implemented using MATLAB. The training was performed on a NVIDIA GeForce GTX 30900 GPU with 24 GB of memory.


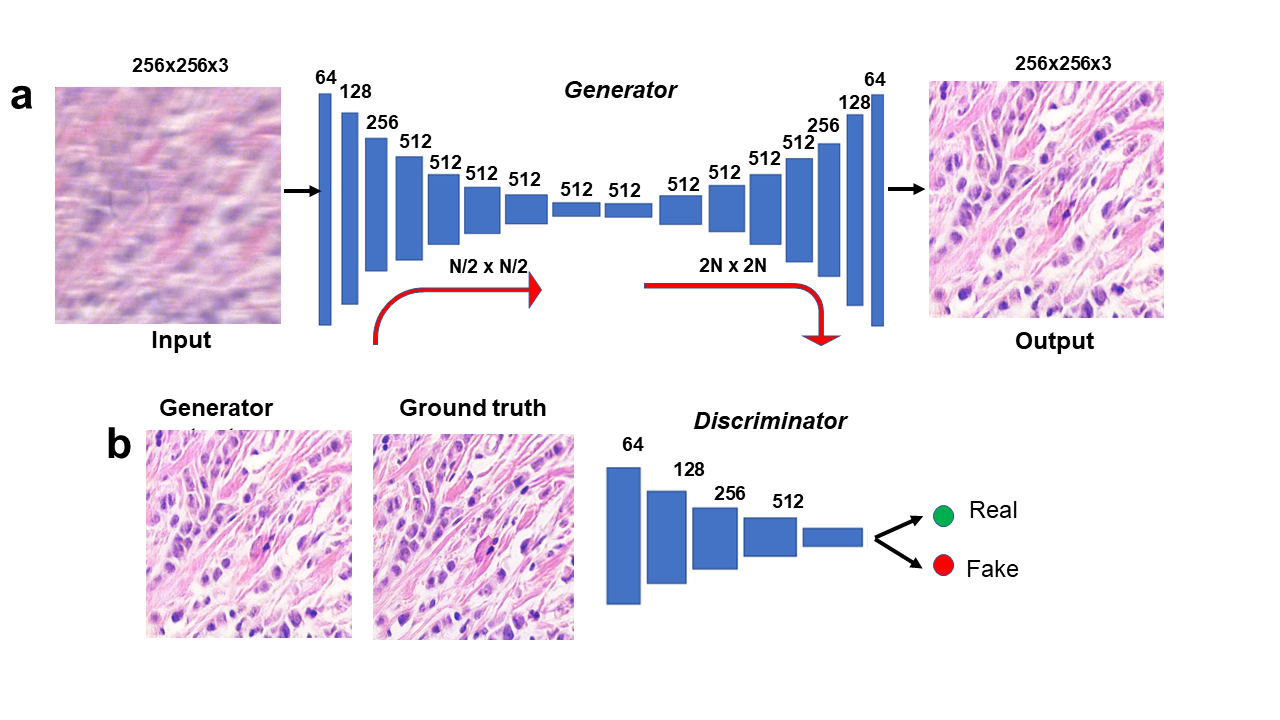


Figure S3. Model architecture. **a** The network consists of a generator model, **b** and a discriminator model, competing with one another. The generator endeavors to render a sharp image and trick the discriminator into judging it as authentic.

**S4. Unseen blood smears:** 50 unseen blood smear images were also reconstructed, a sample of which is shown in Figure S4. GANscan does successfully generates a standard phase contrast image from a highly blurred input. Although some of the cell boundaries are not as smooth and round as in the control data, there is rarely any hallucination of new cell rims.


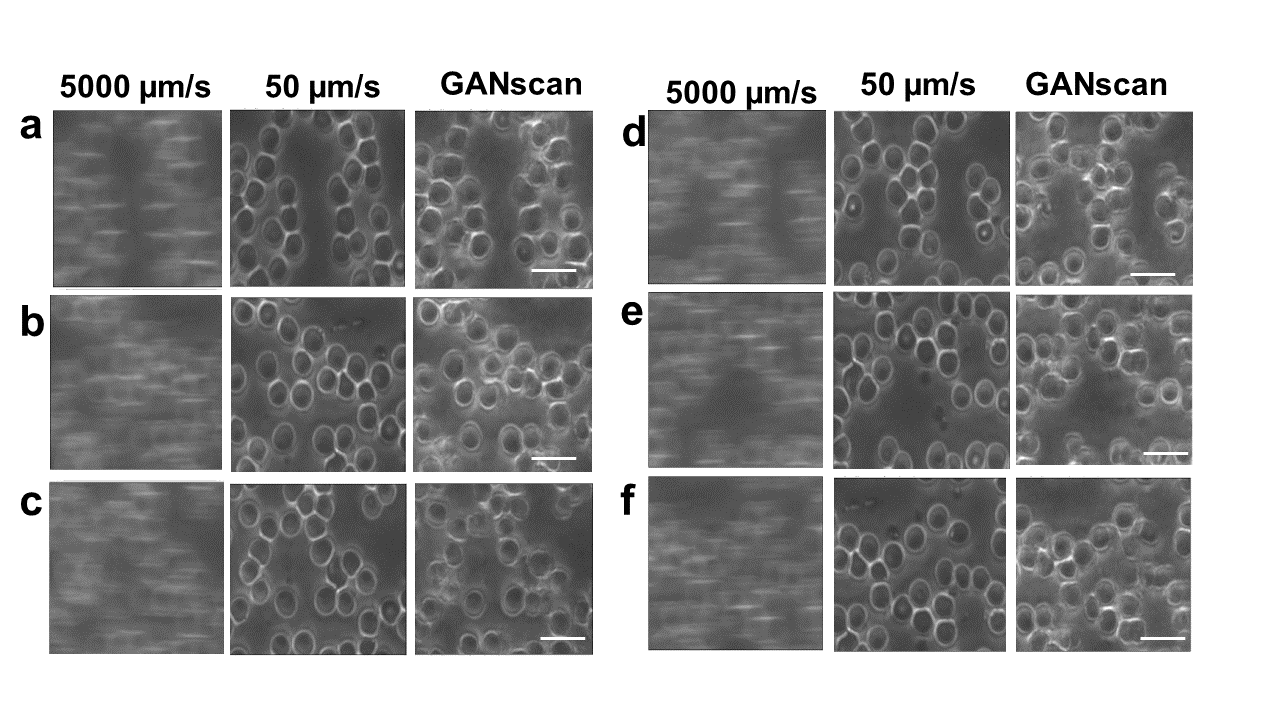


Figure S4. **a-f** Test set conversion of phase contrast unlabeled blood smear images moving at 5000 µm/s with control at 50 µm/s. Scale bar **5 µm.**

**S5-7. Numerical assessments of pathology results:** when comparing the biopsy test sets to their controls, the test sets had an average structural similarity index measure (SSIM) of 0.82 and a mean peak signal-to-noise-ratio (PSNR) of 27. The deconvolution results produced an SSIM and PSRN of 0.71 and 26, respectively, for the identical data set. The blood smear GANscan results have an improved SSIM value of 0.73 compared with the deconvolution value of 0.66 and the same PSNR disparity as in the brightfield case.

The examples of biopsy results in Figure S5 illustrate how the GANscan restorations are superior to the deconvolution operations. The deconvolutions succeed at cancelling out the increased width of features that is caused by the motion blur, however, there is a still a generally diminished resolution. GANscan addresses this shortcoming with particular effectiveness about epithelial areas. Even though this distinction is not wholly conveyed in the SSIM values, the margin of improvement in the GANscan cases are likely due to this resolution enhancement.


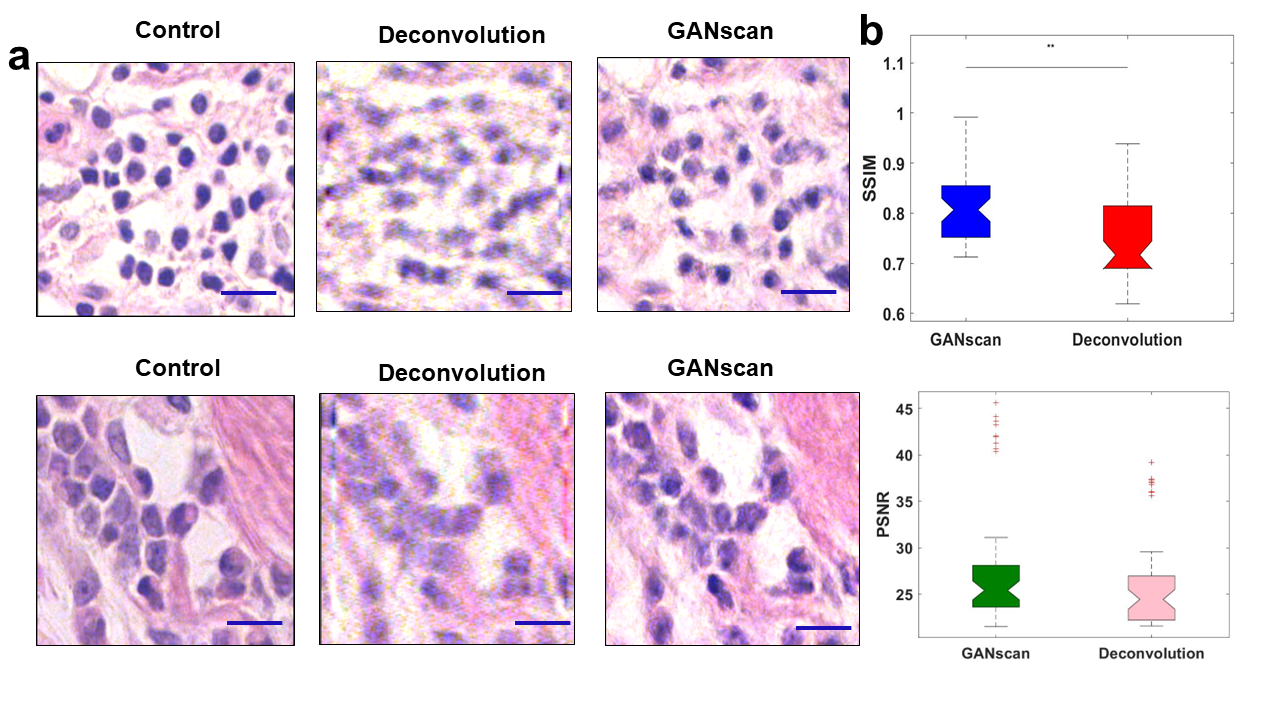


Figure S5. **a** Comparison of examples of deconvolved images with GANscan results, as well as **b** SSIM and PSNR values for the test sets. (**) indicates a p value<0.01. Scale bar **5 µm.**

The biopsy test set corresponding to a different patient achieved a similar average structural similarity index measure (SSIM) of 0.83 and a mean peak signal-to-noise-ratio (PSNR) of 26 when compared against their controls, demonstrating that the GANscan technique is applicable to samples entirely separate from of the training data (Fig S6). For the same dataset, the deconvolution results again gave inferior results of SSIM and PSRN of 0.77 and 25, respectively.

The same evaluations were also performed for the stop-and-stare ground truths set. Figure S7 indicates that there is no statistically significant difference between the values using the stop-and-stare and the 50 μm s^-1^ controls, proving that our pairing strategy is valid.


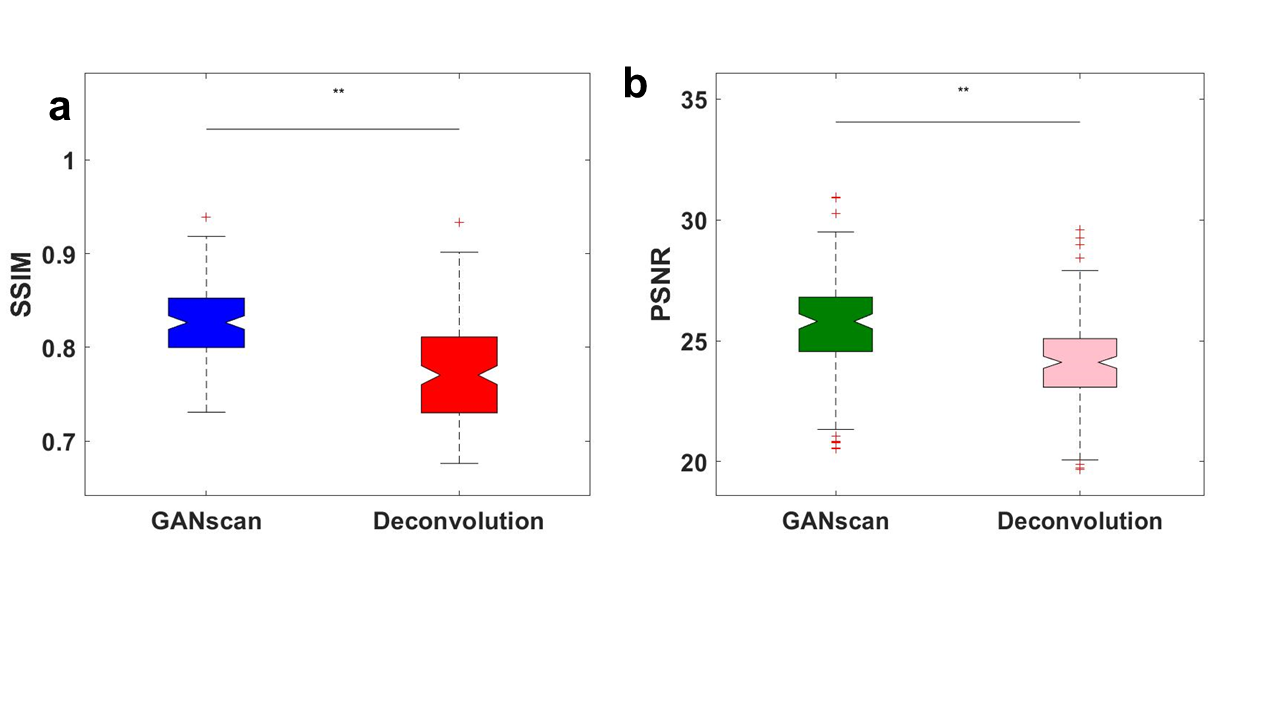


Figure S6. **a** SSIM and **b** PSNR comparisons between GANscan results and Deconvolution for the different patient test set. (**) indicates a p value<0.01.


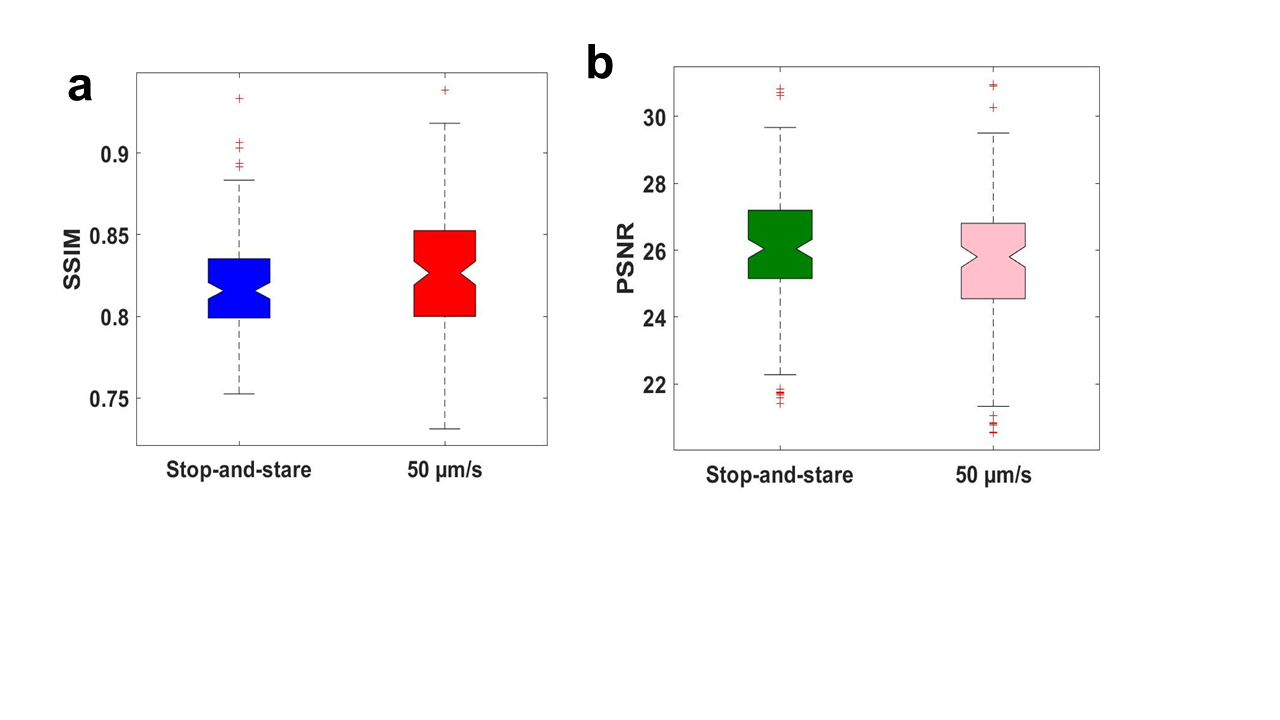


Figure S7. **a** SSIM values for the test sets with comparison of stop-and-stare ground truth with 50 µm/s ground truth. **b** SSIM values for the test sets with comparison of stop-and-stare ground truth with 50 µm/s ground truth.

**S8. Plot profiles:** A second way the pathology results were evaluated was using line sections and plot profiles. Figure S8 shows a sample biopsy image in all three modes with their power spectra and line sections. The brightfield plot profiles indicate a strong overlap between the slow and reconstructed images, whereas the blurry image has a line profile that is blended and diminished in intensity. In the frequency domain, the slow and reconstructed images have broader and higher frequencies, whereas the power spectrum of the blurry image is tighter especially along the horizontal axis, as expected.


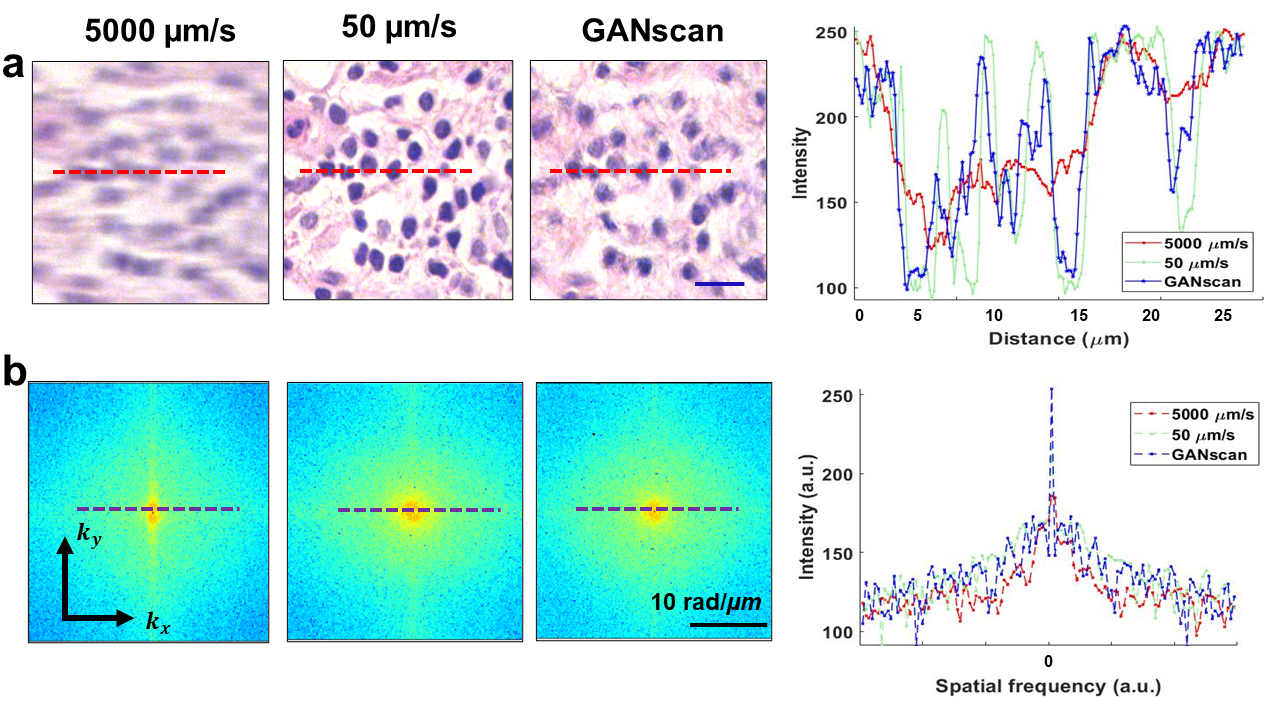


Figure S8. **a** Example test images in fast, slow and reconstructed versions with the intensity plot profiles of the red dash line through the image. **b** The power spectra of a with plot profile of purple dashed line in the images. Scale bar **5 µm.**

**S9. Blood smear evaluations:** the examples of blood smear results in Figure S9 demonstrate how the GANscan restorations are also markedly better to deconvolution operations. The deconvolutions again manage to repair the distorted width of features that is caused by the motion blur, however, there is a still a general haziness in the images. GANscan addresses this imperfection and manages to mimic the correct resolution and clarity of the control micrographs. Even though this difference is not entirely reflected in the SSIM values, the higher scores in the GANscan cases are likely due to this amelioration in structural integrity.


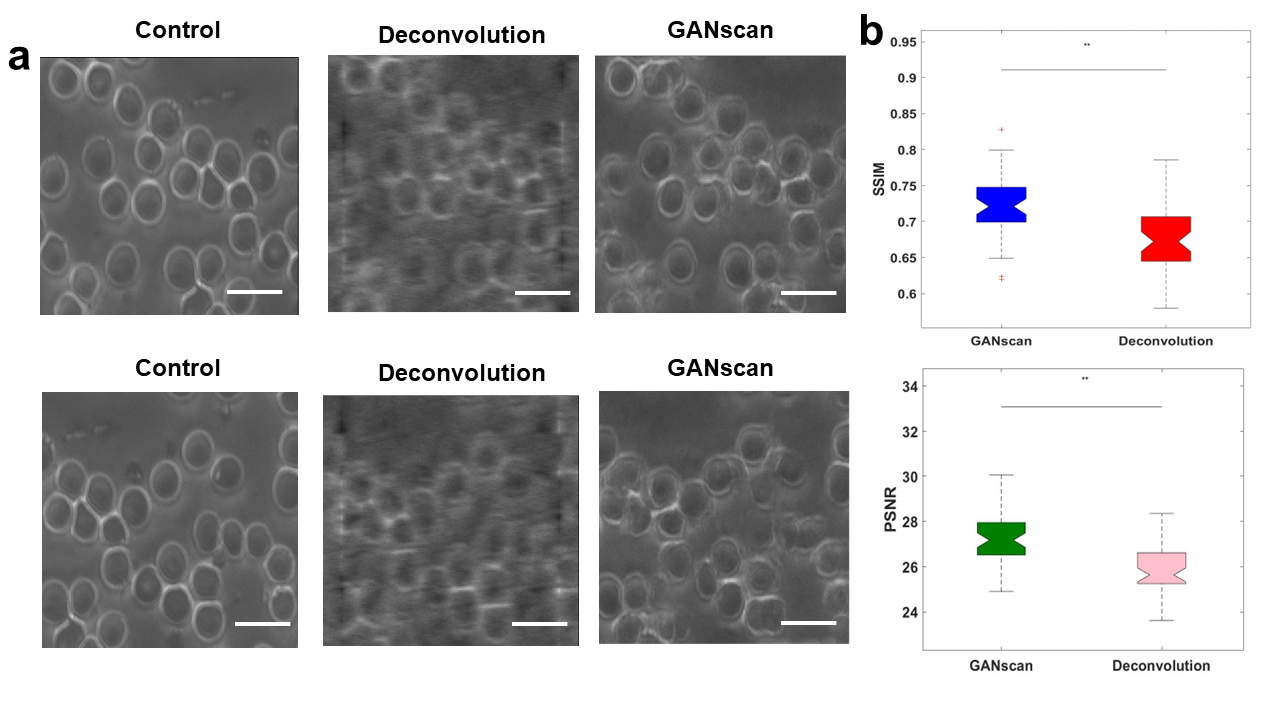


Figure S9. **a** Comparison of examples of deconvolved phase contrast blood smear images with GANscan results, as well as **b** SSIM and PSNR values for the test sets. (**) indicates a p value<0.01. Scale bar **5 µm.**

**S10. Stitching reconstructed images:** biopsy stitches of the moving blurred images were also created (Fig. 6) by concatenating the images horizontally and vertically in their respective scanning order, resulting in a 7 x 15 stitch with a dimension of around 3 mm × 1.5 mm. With such a large FOV, the increase in clarity is less noticeable, but upon closer examination reveals a significant improvement in overall feature delineation. For comparison, stitches for 4,000 μm s^-1^ were also produced (Fig. S10).


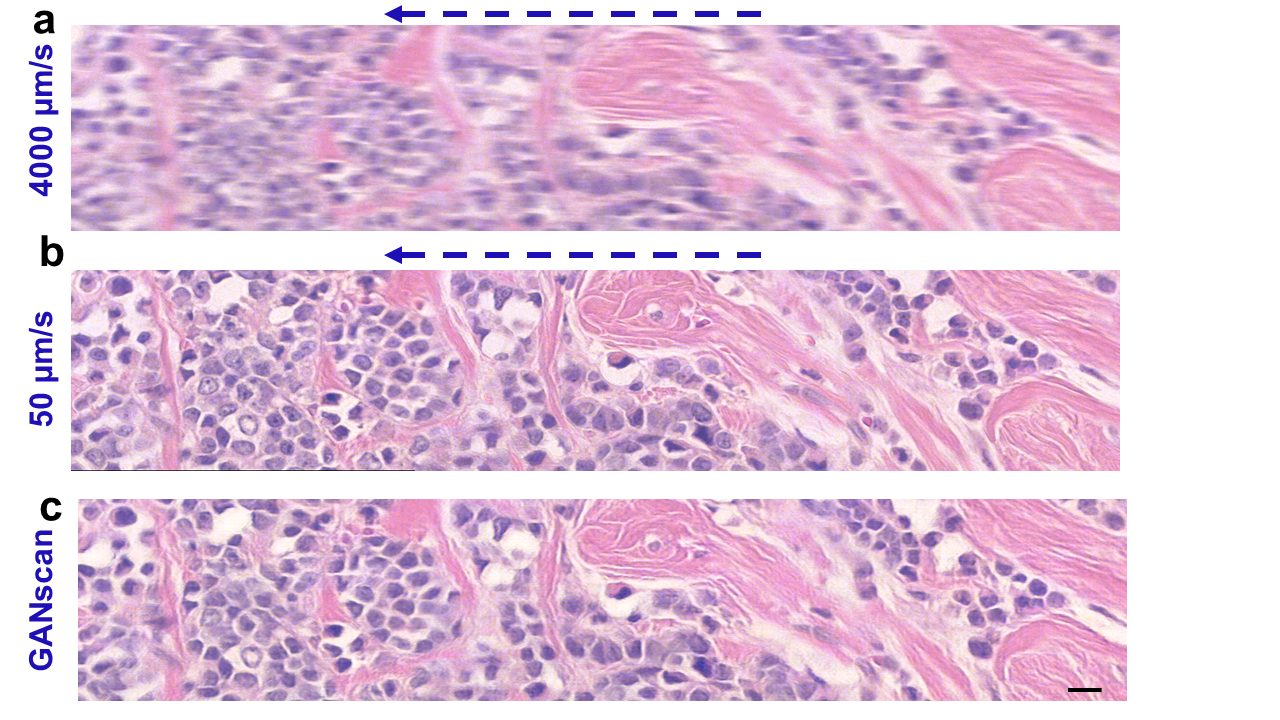


Figure S10. Example of a stitch with training data of **a** blurry, **b** sharp control and **c** reconstructed images. Scale bar **25 µm.**

**SI References**

1. Rivenson, Y., et al., *PhaseStain: the digital staining of label-free quantitative phase microscopy images using deep learning.* Light: Science & Applications, 2019. **8**(1): p. 23.

2. Christiansen, E.M., et al., *In silico labeling: predicting fluorescent labels in unlabeled images.* Cell, 2018. **173**(3): p. 792-803. e19.

3. Ounkomol, C., et al., *Label-free prediction of three-dimensional fluorescence images from transmitted-light microscopy.* Nature methods, 2018. **15**(11): p. 917-920.

4. Barbastathis, G., A. Ozcan, and G. Situ, *On the use of deep learning for computational imaging.* Optica, 2019. **6**(8): p. 921-943.

5. de Haan, K., et al., *Deep learning-based transformation of H&E stained tissues into special stains.* Nature communications, 2021. **12**(1): p. 1-13.

6. Isola, P., et al. *Image-to-image translation with conditional adversarial networks*. in *Proceedings of the IEEE conference on computer vision and pattern recognition*. 2017.
